# Cartilage Oligomeric Matrix Protein Angiopoeitin-1 Provides Benefits During Nerve Regeneration *In Vivo* and *In Vitro*

**DOI:** 10.1007/s10439-015-1342-3

**Published:** 2015-05-27

**Authors:** Longhai Qiu, Bo He, Jun Hu, Zhaowei Zhu, Xiaolin Liu, Jiakai Zhu

**Affiliations:** Department of Orthopedics and Microsurgery, The First Affiliated Hospital of Sun Yat-sen University, No. 58 Zhongshan Road 2, Guangzhou, 510080 China

**Keywords:** Acellular nerve graft, Extracellular matrix, Nerve regeneration, Tissue engineering, Neovascularization

## Abstract

Our group pioneered the study of nerve regeneration in China and has successfully developed human “acellular nerve grafts (ACNGs)”. However, our clinical studies revealed that the effects of ACNGs for long and large nerve defects are far from satisfactory. To improve the efficacy of ACNGs, we combined Cartilage oligomeric matrix protein angiopoietin-1 (COMP-Ang1) with ACNGs in rat sciatic nerve injury models and observed the outcomes *via* angiographic, morphological, and functional analyses. Co-cultures of endothelial cells (ECs) and dorsal root ganglion neurons (DRGs) were also used to characterize the relationship between neovascularization and nerve regeneration. The results showed significant improvements in early neovascularization, nerve regeneration, and functional outcomes *in vivo* in the ACNG + COMP-Ang1 group. *In vitro*, neurite length, and density as well as the expression levels of neurofilament 68 (NF68) and phosphorylated-Tie-2 (p-Tie-2) significantly increased when ECs were co-cultured with DRGs using COMP-Ang1. p-Tie-2 expression dramatically decreased after treatment with a Tie-2 kinase inhibitor (S157701), which consequently decreased the level of NF68. COMP-Ang1 can be concluded to promote early neovascularization followed by brisk nerve regeneration, and the mechanism of this regeneration may involve the modulation of the p-Tie-2 and Tie-2 receptors on ECs. These findings demonstrate that ACNGs can be modified using COMP-Ang1 to improve their efficacy in repairing peripheral nerve defects in clinical trials.

## Introduction

Autologous nerve grafts, the current “gold standard” for the treatment of peripheral nerve defects, suffer from their inherent disadvantages, and a suitable alternative has long been sought after. At present, acellular tissue matrix is an attractive material for tissue repair in biologically derived materials. Our group pioneered the study of nerve regeneration in China and has successfully produced human “acellular nerve grafts (ACNGs)”.^22^ However, our clinical studies revealed that the clinical prognoses of nerve defects are far from satisfactory, especially for long and large nerve defects. A meta-analysis of our previous studies showed that graft length is an independent predictor of prognosis, and longer defects indicate poorer recovery.^23^ Improving the efficacy of ACNGs for short and long nerve defects is a major issue in current research. Previous studies demonstrated that the rate of axonal regeneration was increased near blood vessels, suggesting an interaction between regenerating axons and blood vessels.^44^ Thus, numerous studies focused on revealing the interaction between neovascularization and nerve regeneration using growth factors,^25^,^39^,^40^ angiopoietins,^30^,^31^ and Schwann cell (SC)/endothelial cell (EC) co-cultures.^17^ The vascular and neural systems are architecturally similar yet execute distinct functions. They share common molecular pathways that regulate cell fate during development.^5^ For example, the branching patterns of peripheral sensory nerves and small skin arteries are related. SCs release vascular endothelial growth factor (VEGF), which instructs blood vessels to branch along the nerves.^38^ Therefore, the relationship between vascular biology and neuroscience helps us to discover new mechanistic insights and therapeutic opportunities.^5^

VEGF and angiopoietin-1 (Ang1) are both potent pro-angiogenic factors that can efficiently prompt vessel formation. Hobson *et al*.^25^ found that VEGF can enhance intraneural angiogenesis and improve nerve regeneration after axotomy, while Ang1, based on its angiogenic properties, has been proposed as a therapeutic strategy to target microvascular diseases in several experimental models.^30^,^31^,^41^ However, the use of Ang1 is limited due to its short working duration. Recently, cartilage oligomeric matrix protein angiopoeitin-1 (COMP-Ang1) has been developed as a soluble, stable, and potent Ang1 variant.^28^,^32^ COMP-Ang1 was generated by replacing the N-terminal portion of Ang1 with the short coiled-coil domain of cartilage oligomeric matrix protein.^7^,^26^ This new protein more potently phosphorylates the Tie-2 receptor than native Ang1, which allows the receptor to function more effectively. Long-term and sustained exposure to COMP-Ang1 was shown to facilitate long-lasting vascular enlargement and blood flow enhancement.^9^ Additionally, COMP-Ang1 is more actively involved in EC migration, pericyte recruitment, and blood vessel formation, remodeling and maturation than the native protein.^6^,^21^ Akt and p38 MAPK phosphorylation have been shown to be important mediators downstream of Ang1 and are involved in angiogenesis and axon growth.^8^,^19^ This study aimed to investigate the interactions between nerve regeneration and neovascularization after the administration of COMP-Ang1 to rats with a sciatic nerve injury. Moreover, ECs and dorsal root ganglion neurons (DRGs) were co-cultured to investigate the mechanism by which COMP-Ang1 prompts neovascularization and nerve regeneration.

## Materials and Methods

### Sciatic Nerve Defect Repaired by ACNG Combined with COMP-Ang1 (*In Vivo*)

Our study was approved by the Ethics Committee of the Medical College of the First Affiliated Hospital of Sun Yat-sen University. The review number is [2013] A-055. This study included 151 healthy female adult Sprague–Dawley (SD) rats (average weight 200–250 g) from the Experimental Animal Center of the First Affiliated Hospital of Sun Yat-sen University. The rats were given a standard diet and cared for with standard procedures.

### Preparation of ACNGs

Fifty healthy rats were selected as donors for the nerve allografts. These rats were anesthetized with an intraperitoneal injection of 10% chloral hydrate (0.3 mL/100 g body weight) (Sinopharm Chemical Reagent Co., Ltd., Shanghai, China). The bilateral sciatic nerves (≥20 mm in length) were excised from the rats, cleaned of external fat and connective tissue and immediately placed in sterile phosphate-buffered saline (PBS). The nerve segments were treated with chemical detergents to remove cellular components as previously described.^22^,^46^ Briefly, the nerve segments were agitated in deionized distilled water for 7 h and treated with 3% (v/v) Triton X-100 (Sigma-Aldrich, St. Louis, MO, USA) overnight, followed by treatment with 4% (w/v) sodium deoxycholate (Sigma-Aldrich, St. Louis, MO, USA) for an additional 24 h. After thorough washing in 10 mM PBS, the extracted acellular nerves were subjected to cobalt-60 irradiation (25 kGy g radiation) for 12 h (Guangzhou Huada Biological Technology Co., Ltd., Guangzhou, China) and stored in 10 mM PBS at 4 °C until use.

### Surgical Procedure

A total of 90 rats (200–250 g) survived the experiment and were randomly divided into two groups: a control group (ACNG group) and a test group (ACNG + COMP-Ang1 group). Each group consisted of 45 animals. All surgical procedures were performed by the same surgeon under aseptic operating conditions using 10% chloral hydrate anesthesia (0.3 mL/100 g body weight). The incision was made on the lateral side of the left thigh to expose the sciatic nerve. With the aid of a surgical microscope, a 10-mm segment of the nerve was removed and microsurgically repaired using a 12-mm ACNG. Penicillin G (10,000 U) (Sigma, Deisenhofen, Germany) was postoperatively injected into the quadriceps femoris for 3 days. In the ACNG + COMP-Ang1 group, all rats received daily intraperitoneal injections of COMP-Ang1 (5 *μ*L/g body weight, Enzo Life Sciences, Farmingdale, NY, USA), while rats in the ACNG group received daily doses of a 0.9% NaCl solution (5 *μ*L/g body weight, i.p.). All rats were housed in cages in a warm environment and permitted free access to food and water.

### Evaluation of Neovascularization

Lead oxide (Pb_3_O_4_, Chengdu Jinshan Chemical Reagent Co., Ltd., Chengdu, Sichuan, China) (50%, w/v) was mixed with a 5% (w/v) gelatin solution and kept in a 40 °C water bath. Five rats in each group were randomly selected at specific time intervals: 7, 14, and 21 days after surgery. The rats were anesthetized with an intraperitoneal injection of 10% chloral hydrate (0.3 mL/100 g body weight). The carotid artery was exposed, and the perfusion agents (10 mL/200 g body weight) were injected into the artery after arterial catheterization. Perfusion was stopped after discoloration of the sclera and extremities was noted. A one-step injection of the perfusion agent was ensured throughout the perfusion process. After successful systemic perfusion, the bilateral sciatic nerves of the rats were carefully exposed and dissected.^48^ Subsequently, 15 mm of nerve tissue was harvested from the inferior margin of the piriformis and fixed in 4% paraformaldehyde for 30 min, then treated with 30% sucrose for 3 h. The harvested nerves were scanned by microCT (ZKKS-MCT-III, Guangzhou Zhongke Kaisheng Medical Technology Co., Ltd., Guangzhou, Guangdong, China) (voltage, 40 kV peak; power, 45 W; four frames every 0.72° of rotation; total rotation range, 360°; total scanning duration, 28 min). The acquired image information was input into Mimics 10.0 software to observe the state of visualization for each aspect of the specimen and establish a visual observation model.

The microvessel volume (mm^3^) and total volume of peripheral nerves (mm^3^) were measured, and the vascular index (VI) and volume fracture (VF) were calculated. The VI was calculated using the following formula: total area of vascular development region/cross-sectional area of nerves × 100%. Volume fracture refers to the vascular content per unit area in the peripheral nerves to be tested and was calculated using the following formula: total microvascular volume of the inspected nerves/total volume of the inspected nerves × 100%. Data are expressed as the mean ± standard deviation. SPSS 13.0 (SPSS, Inc., Chicago, IL, USA) was used to perform the independent *t* test. Statistical significance was set at *p* < 0.05.

### Molecular Biology Evaluation During Nerve Regeneration

On days 14, 21, and 28, rats in each group were randomly selected and sacrificed to harvest the nerve grafts (*n* = 5). All nerves were fixed in 4% paraformaldehyde for 24 h at 4 °C, followed by dehydration in a 20% sucrose solution for 24 h and a second dehydration in 30% sucrose solution for another 24 h.

The nerve samples were serially sectioned at a thickness of 5 µm on a freezing microtome (Leica CM3050S; Leica, Wetzlar, Germany) and collected on superfrost/plus slides (Cytotest; Jiangsu Cytotest Experimental Supplies Co., Ltd., Haimen, Jiangsu, China). For each sample, ten sections per animal were randomly selected from the medium part. Immunofluorescence studies were performed using a monoclonal anti-neurofilament 200 (NF200) (Sigma-Aldrich, N4142, USA, 1:200). The staining procedure was performed as described in previous studies.^43^,^46^ Images of the stained sections were captured with a fluorescence microscope attached to a CCD spot camera (DFC350FX/DMIRB; Leica). Neurofilament expression was measured based on the integral optical density (IOD) of the NF200-immunoreactive area using Image Pro Plus 6.0 (IPP) software (Media Cybernetics, MA). The images of graft segment were converted to gray scale 8 using IPP, and the color range was selected. Finally, the computerized optical density representing neurofilament expression was calculated using the measure and count function. Data are expressed as the mean ± standard deviation. SPSS 13.0 was used to perform the independent *t* test. Statistical significance was set at *p* < 0.05.

Eight weeks after the operation, rats were selected from each group and anesthetized for neuronal tracing (*n* = 5). The left sciatic nerve was carefully exposed and separated from the fascia and muscle. Ten microliters of 2% fluoro-gold was injected into the rat sciatic nerve trunk at a point distal to the grafts; the axons were then allowed to absorb the solution. After 14 days, the rats were sacrificed to harvest their dorsal root ganglions and spinal cord.^13^ For each sample, 5 sections (16 µm thickness) of ganglion and spinal cord (20 µm thickness) were randomly selected and mounted on glass slides. Neurons labeled with FG were counted in each ganglion or spinal cord section under a fluorescence microscope. Ten high-power fields per specimen were randomly selected and analyzed with IPP. SPSS 13.0 was used to perform the independent *t* test. Statistical significance was set at *p* < 0.05.

The gastrocnemius muscles were simultaneously harvested for hematoxylin and eosin (HE) staining, and the graft segments were harvested for transmission electron microscopy (*n* = 5).^47^ The muscle samples were post-fixed with formalin, embedded in paraffin, and sectioned transversely (4 µm thickness). For each sample, five sections of gastrocnemius (4 µm thickness) were randomly selected. The sections were deparaffinized, rehydrated in an ethanol series, and stained with HE. The ultrathin sections were stained with lead citrate and uranyl acetate and then examined under a Philips CM120 transmission electron microscope equipped with an image acquisition system at ×8000 magnification to measure the thickness of the myelin sheaths. Photographs from ten random fields of each ultrathin nerve section were analyzed using IPP.

At 12 weeks, toluidine blue (Chengdu Jinshan Chemical Reagent Co., Ltd., Chengdu, Sichuan, China) staining was performed as previously described.^47^ Briefly, nerve graft segments were harvested and quickly immersed in 2.5% Na–cacodylate-buffered glutaraldehyde solution for 2 h, fixed for 2 h in 2% Na–cacodylate-buffered osmium tetroxide, serially dehydrated in increasing concentrations of ethanol, infiltrated with and embedded in Epon 812 (Ted Pella, Redding, CA, USA), sectioned (4 *μ*m thickness), and finally stained with toluidine blue to evaluate the efficacy of nerve regeneration. Transection was performed at the middle of the nerve graft segments. The average number of myelinated axons and the fiber diameter were analyzed using an Olympus BX60 microscope and IPP software. For each sample, photographs of three random fields were taken and analyzed to measure the number of axons and their diameters. SPSS 13.0 was used to perform the independent *t* test. Statistical significance was set at *p* < 0.05.

### Functional Evaluation of the Sciatic Nerve

The sciatic functional index (SFI) was tested 2, 4, 6, 8, 10, and 12 weeks after the surgery.^11^ The rats of each group (*n* = 5) were placed in a confined walkway with a dark shelter at the end, and the investigators were blinded to the animal treatment groups during the walking track analysis. The electrophysiological evaluation was performed 12 weeks after the surgery. The injured nerves were exposed after anesthetization. Electrical stimuli were applied to the nerve trunks at the distal or proximal portions of the grafting site, and the compound muscle action potentials (CMAPs) and nerve conduction velocity (NCV) were recorded on the triceps surae muscle belly using a portable biological function experimental system (BL-420F; Tai Meng, Inc., Chengdu, China). Data are expressed as the mean ± standard deviation. SPSS 13.0 was used to perform the independent *t* test. Statistical significance was set at *p* < 0.05.

### Observation of the Interaction Between Angiogenesis and Nerve Regeneration (*In Vitro*)

A human umbilical vein endothelium-derived cell line, ECV304, was obtained from a lab at the Cell Culture Experimental Animal Center of Sun Yat-sen University. Dorsal root ganglions were harvested from 3-day-old postnatal SD rats according to established procedures.^31^

The ganglions were obtained from 6 SD neonates and cultured in flasks with Dulbecco’s modified Eagle medium (DMEM)/F-12 medium (Gibco, USA) containing 10% fetal bovine serum (FBS, Gibco, USA), 1% penicillin–streptomycin solution (Gibco, USA), 2 mM forskolin (Sigma-Aldrich, Tokyo, Japan), and 100 ng/mL nerve growth factor (NGF, Sigma-Aldrich, USA). The flasks were kept in a humidified atmosphere of 5% CO_2_ in air at 37 °C for 24 h. After being cultured with the medium containing 10% fetal bovine serum and NGF, all cells grew slowly. The neurotrophic effects of NGF increased the viability of neurons to accelerate their growth, while the low serum concentration inhibited the activities of other cells in dorsal root ganglions, including fibroblasts and SCs. The DRGs were divided into five groups, group A (DRGs), group B (DRGs treated with 100 ng/mL COMP-Ang1), group C (ECV304/DRGs co-culture), group D (ECV304/DRGs co-culture treated with 100 ng/mL COMP-Ang1), and group E (ECV304/DRGs co-culture treated with 100 ng/mL COMP-Ang1 + 250 nM Tie-2 kinase inhibitor S157701 (Sellect, Houston, USA)). The cells were trypsinized and plated in a 24-well plate at a density of 2 × 10^5^ cells per well for the former two groups and 1 × 10^5^ cells per well for the latter three groups. The cells were cultured for 24 h before co-culture. The ECV 304 cells were thawed and plated in a Transwell (0.4 µm pore size, Millipore, USA) at a density of 1 × 10^5^ cells per well. The Transwell layer was placed on top of the confluent layer of DRGs for the co-culture groups. In group E, 250 nM S157701 was added to the well, and the cells were cultured for 1 h. The groups were then subjected to different conditions (listed above in the bracket after the different groups) for 72 h.

All specimens were fixed with 2% paraformaldehyde (dissolved in 0.01 M PBS) for 30 min and then dehydrated in 5% sucrose at 37 °C for 15 min. This step was followed by incubation with 10% normal goat serum for 1 h. Subsequently, monoclonal mouse anti-NF200 antibody (Sigma-Aldrich, USA, 1:200) and monoclonal rabbit anti-β-tubulin antibody (Sigma-Aldrich, USA, 1:300) were added to the specimens, which were then incubated with the cells at 4 °C overnight. Goat anti-mouse antibody (Cell Signaling Technology, USA, 1:400) and goat anti-rabbit antibody (Cell Signaling Technology, USA, 1:500) were then added, and the samples were incubated for 1 h. Lastly, the samples were incubated with 2 *μ*g/mL DAPI (Merck, Germany) for 10 min.

The neurite length and number of perikarya per 0.5-mm area were assessed as established in previous studies.^31^ To assess neurite length, a 400-*μ*m diameter circle was drawn around a single neuron, and the total length of NF200-positive neurites (including first and higher orders) lying within the circle was measured. Thirty neurons were studied per group, and each experiment was repeated three times. To calculate the number of perikarya, five high-power fields were randomly selected in one culture well. The NF200-positive neurons in a 0.5-mm^2^ area were then counted. The cells were lysed in RIPA buffer (1× PBS, 1% NP40, 0.5% sodium deoxycholate, 0.1% sodium dodecyl sulfate, and the protease inhibitor cocktail Roche) with phenylmethanesulfonyl fluoride on ice for 30 min and then centrifuged for 10 min at 15,000×*g* and 4 °C to extract protein. The protein samples were separated by sodium dodecyl sulfate polyacrylamide gel electrophoresis (10% Bis–Tris gel), transferred to polyvinylidene fluoride membranes (Millipore, Bedford, USA), and blocked with 5% bull serum albumin for 1 h, followed by incubation with mouse anti-neurofilament 68 (NF68) (Cell Signaling Technology, USA, 1:1000), β-actin (Sigma-Aldrich, USA, 1:1500), rabbit anti-Tie-2 (Cell Signaling Technology, USA, 1:1000), or rabbit anti-phosphorylation-Tie-2 (Cell Signaling Technology, #4226, USA, 1:1000) antibody at 4 °C overnight. After washing, the membrane was incubated with peroxidase-conjugated secondary antibody for 1 h at room temperature, washed in Tris-buffered saline containing tween and developed using an enhanced chemiluminescence system (Millipore, USA). SPSS 13.0 was used to perform one-way ANOVA. Statistical significance was set at *p* < 0.05.

## Results

### Relationship Between Neovascularization and Nerve Regeneration (*In Vivo*)

#### Qualitative and Quantitative Evaluation of Neovascularization

**Figure 1:**
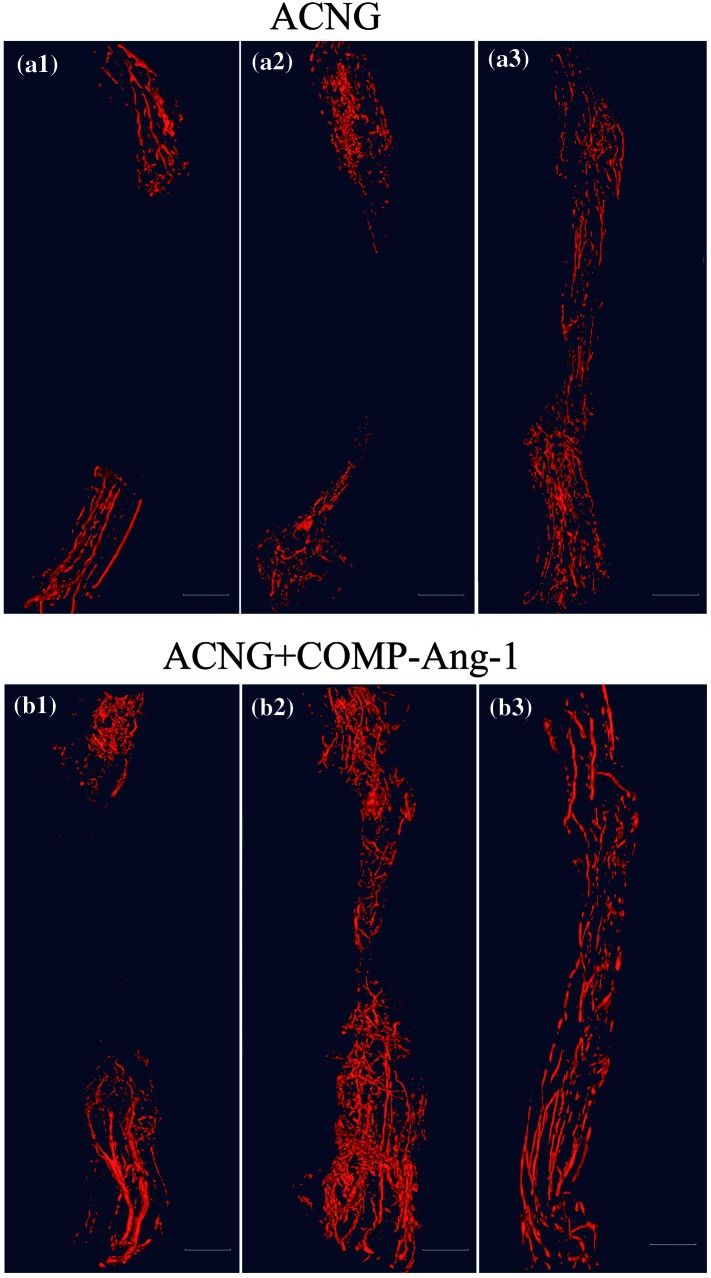
3D-vessel reconstruction of grafts at 7 (a1, b1), 14 (a2, b2) and 21 days (a3, b3) after surgery. The 3D vascular study indicated that vessels had already recanalized in the ACNG + COMP-Ang1 group but not in the ACNG group at 14 days. Scale bars = 100 *μ*m.

**Figure 2:**
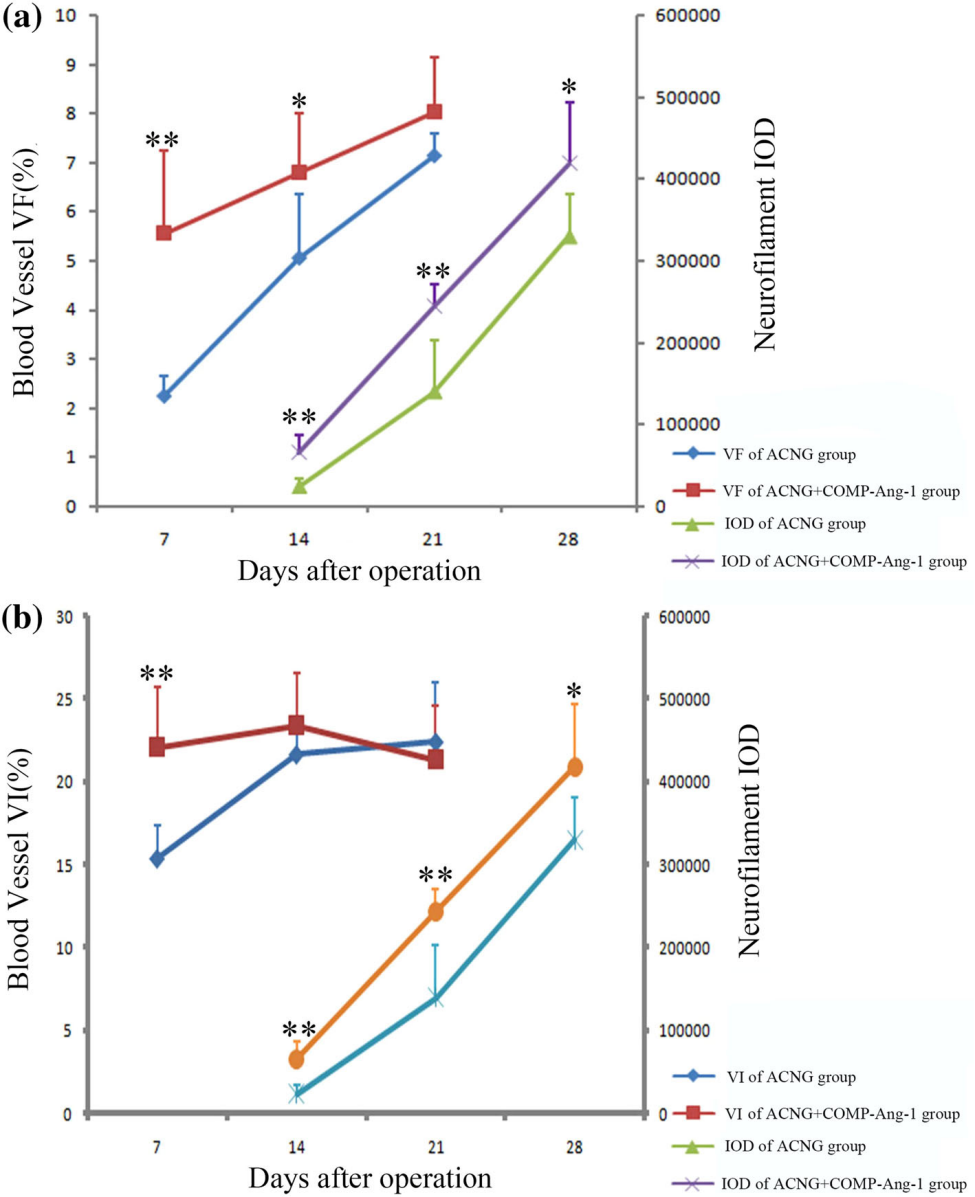
Relationship between neovascularization and nerve regeneration in terms of the blood vessel VF, blood vessel VI and neurofilament IOD. Both VF (day 7 and day 14) and VI (day 7) were higher in the ACNG + COMP-Ang1 group than in the ACNG group. The IOD was greater in the ACNG + COMP-Ang1 group than in the ACNG group throughout the experiment (a and b). **p < 0.01 and *p < 0.05. Mean ± SD.

Figure 1 shows the three-dimensional (3D) vessel reconstruction of grafts 7, 14, and 21 days after surgery. After 14 days, the vessels had already recanalized in the ACNG + COMP-Ang1 group but not in the ACNG group. Both the VF and VI were higher in the ACNG + COMP-Ang1 group than in the ACNG group on days 7 and 14. COMP-Ang-1 yielded VF values of 5.56 ± 1.67% vs. 2.24 ± 0.42 (*p* < 0.05) on days 7, 6.78 ± 1.22% vs. 5.05 ± 1.31% (*p* < 0.05) on days 14, while VF did not significantly differ between these two groups after 21 days (8.02 ± 1.11% vs. 7.13 ± 0.46%, *p* > 0.05). COMP-Ang-1 yielded VI values of 22.31 ± 1.89% vs. 15.27 ± 0.80% (*p* < 0.05) on days 7, while VI did not significantly differ between these two groups after 14 days (23.30 ± 3.31 vs. 21.68 ± 1.52, *p* > 0.05) and 21 days (21.35 ± 3.24 vs. 22.43 ± 3.61, *p* > 0.05) (Figs. 2a and 2b).

#### Morphological Evaluation of Nerve Regeneration

**Figure 3:**
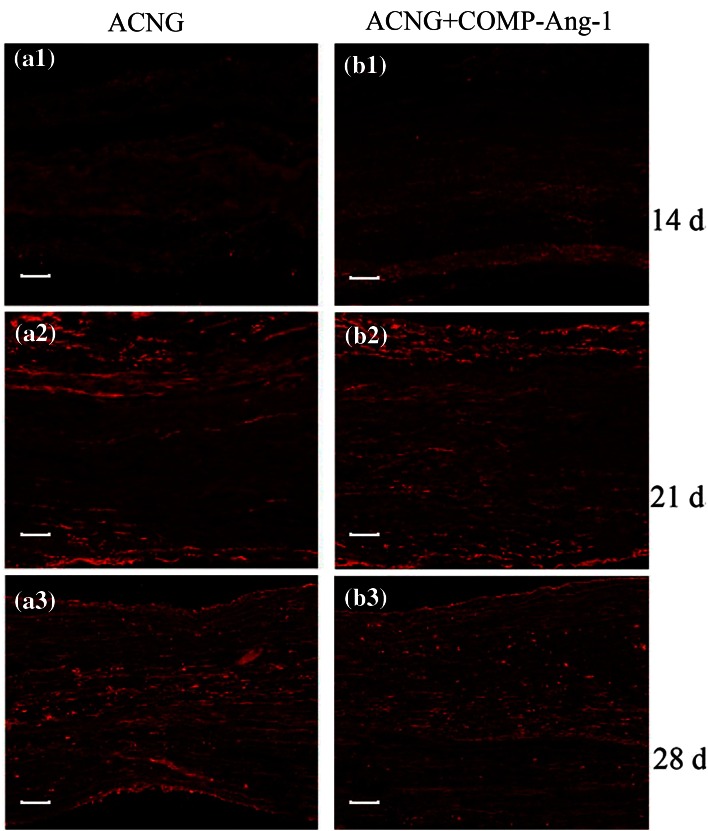
NF200 immunoreactivity of the ACNG group and the ACNG + COMP-Ang1 group at 14 days (a1, b1), 21 days (a2, b2) and 28 days (a3, b3) after surgery. Almost no NF200 expression was found in the ACNG group at 14 days, and the NF200-positive cells in the ACNG + COMP-Ang1 group were distributed sporadically 14 days after surgery. At 21 and 28 days, neurofilaments could be identified in both groups, and the NF200-positive cells increased more quickly in the ACNG + COMP-Ang1 group than in the ACNG group. Scale bars = 50 *μ*m.

**Figure 4:**
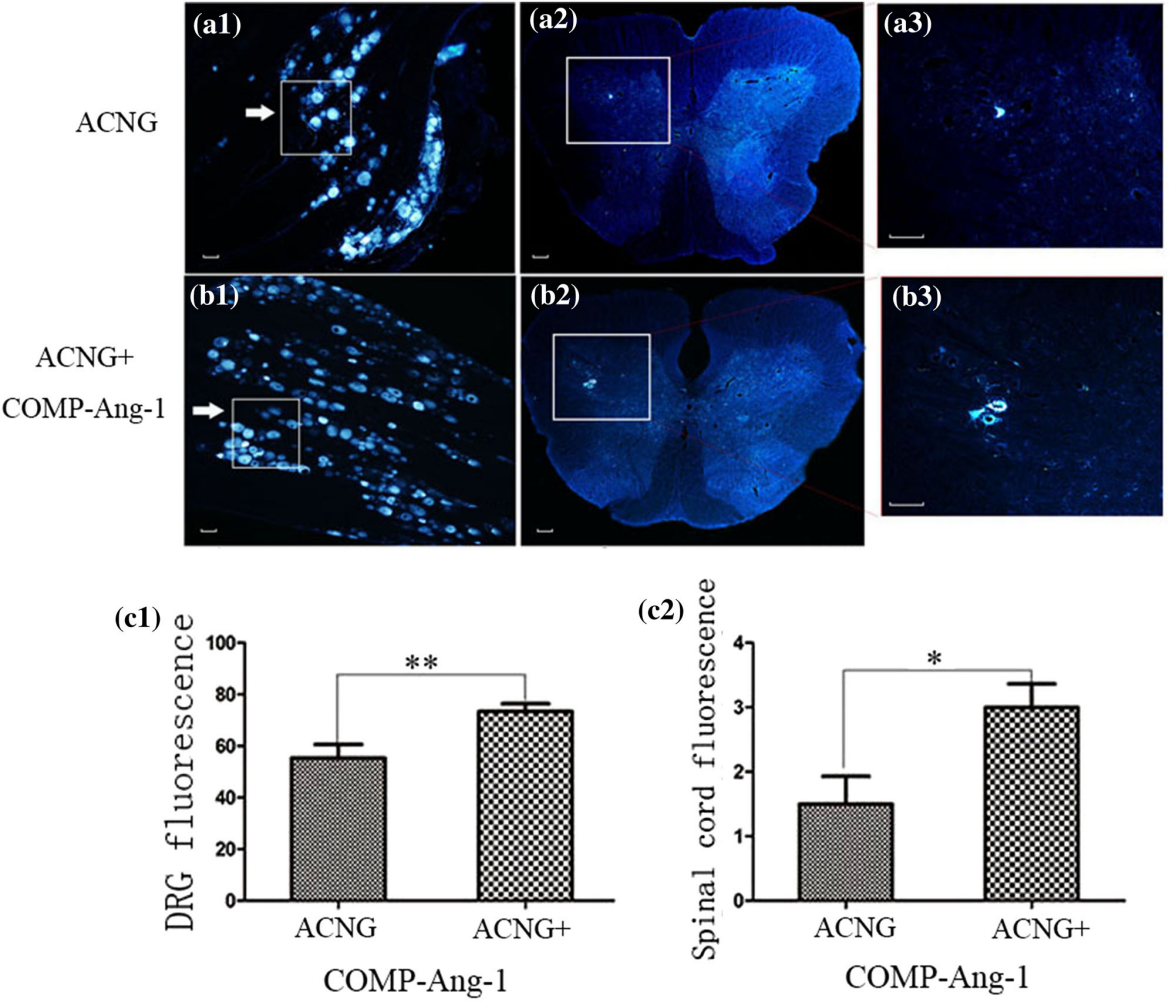
FG retrograde tracing to examine the reverse transportation capability of neurons. The density of FG-labeled sensory neurons is indicated by an arrow in the dorsal root ganglions (a1, b1), and the motor neurons are indicated by an arrow in the spinal cord (a2, b2). DRGs (Figs. 4a1 and 4b1) and motor neurons (Figs. 4a2 and 4b2) fluoresced more strongly in the ACNG + COMP-Ang1 group than in the ACNA group, indicating that COMP-Ang1 can increase retrograde transport in the regenerating nerves. Magnified photographs of a2 (a3) and b2 (b3). Scale bars = 50 *μ*m.

**Figure 5:**
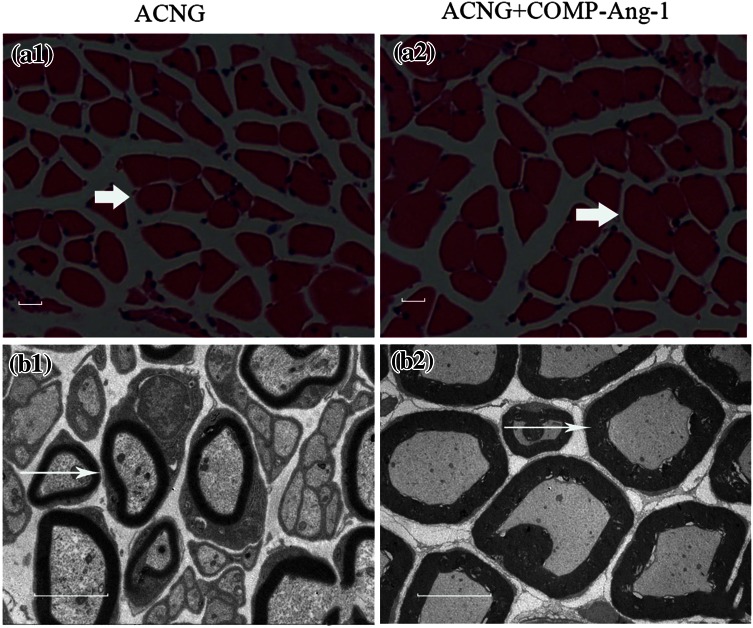
H&E staining of gastrocnemius muscle and ultrastructure of sciatic nerves. Arrows show thicker and more compact myofibers of the gastrocnemius in the COMP-Ang1 group (a2) compared with the control group (a1). Electron microscopy showed well-organized axons and thicker myelination in the COMP-Ang1 group (b1, b2). Scale bars = 50 *μ*m.

Obvious changes in the NF200 protein levels could be observed in both groups on days 14, 21, and 28 (Fig. 3). However, the IOD was higher in the ACNG + COMP-Ang1 group than in the ACNG group, with values of 114,000 ± 21,596 vs. 15,166 ± 10,107 (*p* < 0.05), 243,833 ± 26,407 vs. 139,833 ± 62,703 (*p* < 0.05), and 418,333 ± 74,944 vs. 330,166 ± 51,615 (*p* < 0.05) on days 14, 21, and 28, respectively (Figs. 2a and 2b). Eight weeks after surgery, FG retrograde labeling revealed that more axons crossed the grafts in the ACNG + COMP-Ang1 group than in the ACNG group. Dorsal root ganglions (Figs. 4a1 and 4b1) and motor neurons (Figs. 4a2 and 4b2) in the ACNG + COMP-Ang1 group fluoresced more strongly than those in the ACNG group, with values of 73.33 ± 7.39 vs. 55.33 ± 12.72 (*p* < 0.05) and 3.00 ± 0.89 vs. 1.50 ± 0.43 (*p* < 0.05), respectively, indicating that COMP-Ang1 increased number of axons labeled with retrograde transport dye (Figs. 4c1 and 4c2). H&E staining of the gastrocnemius muscles in the ACNG + COMP-Ang1 group demonstrated thicker and more compact myofibers (Figs. 5a1 and 5a2). In addition, the nerve ultrastructure observed *via* transmission electronic microscopy demonstrated that the nerves in the ACNG + COMP-Ang1 group had more myelinated axons and thickened sheaths, and they appeared more organized than to those of the ACNG group (Figs. 5b1 and 5b2).

**Figure 6:**
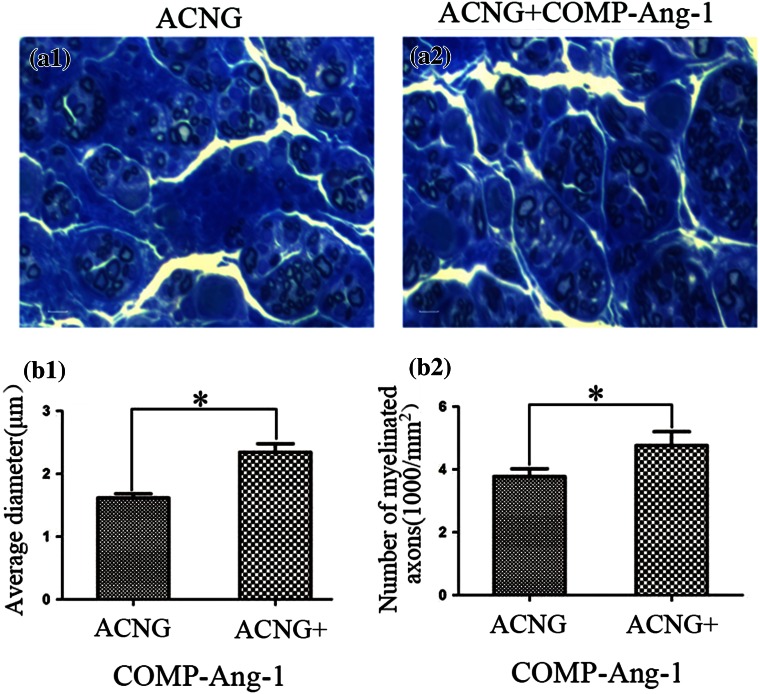
Toluidine blue staining (transverse sections) of the harvested grafts from the ACNG group (a1) and the ACNG + COMP-Ang1 group (a2) 12 weeks after nerve grafting. Compared with the ACNG group, the neural architecture was more organized in the ACNG + COMP-Ang1 group in terms of the average diameter of the nerve (b1) or the number of myelinated axons (b2). *p < 0.05. Error bars correspond to the mean ± S.D. Scale bars = 50 *μ*m.

After 12 weeks, toluidine blue staining (transverse sections) showed that the nerve grafts in the ACNG + COMP-Ang1 group were more compact, uniform, and organized in structure than those in the ACNG group. The axons significantly differed between the two groups, with a higher average diameter (2.34 ± 0.30 vs. 1.76 ± 0.15 *μ*m, *p* < 0.05) and more myelinated axons (4860 ± 870 vs. 3780 ± 540/mm^2^, *p* < 0.05) in the ACNG + COMP-Ang1 group than in the ACNG group. The nerve grafts in the ACNG + COMP-Ang1 group were more compact, uniform and organized in structure than those in the ACNG group (Fig. 6).

#### Functional Evaluation of Nerve Regeneration

**Figure 7:**
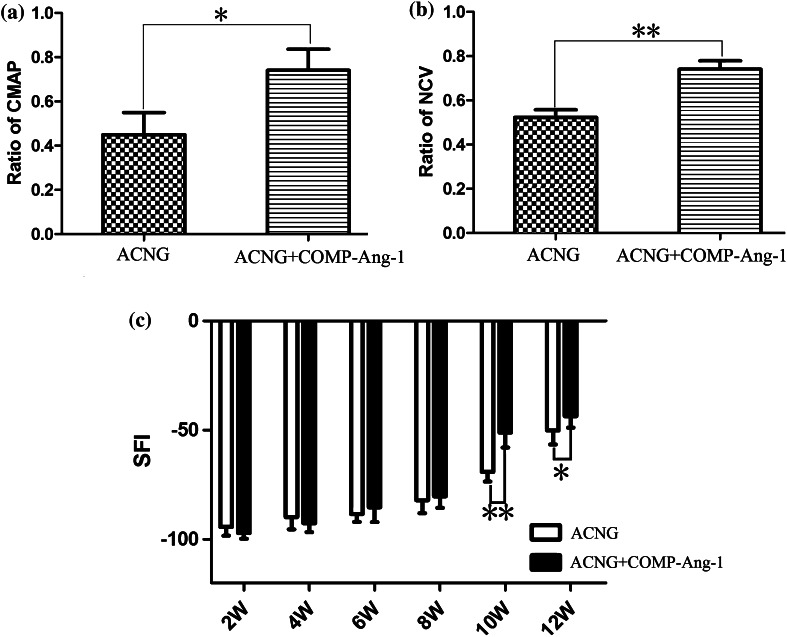
Electrophysiological evaluation of nerve regeneration. Significantly increased CMAPs (a), NCV (b), and SFI (c) in the ACNG + COMP-Ang1 group indicated that COMP-Ang1 improved the functional recovery of the transected sciatic nerve. *p < 0.05, **p < 0.01. Error bars correspond to the mean ± SD.

The SFI was calculated every 2 weeks (2, 4, 6, 8, 10, and 12 weeks) after surgery. Compared to the ACNG group, significant improvement could be observed at 10 weeks (51.33 ± 4.41 vs. 69.33 ± 6.78, *p* < 0.05) and 12 weeks (43.67 ± 5.20 vs. 50.17 ± 6.40, *p* < 0.05) in the group that received COMP-Ang1. The CMAPs increased in the ACNG + COMP-Ang1 group (0.74 ± 0.10 vs. 0.45 ± 0.10, *p* < 0.05) accompanied by a higher NCV (0.74 ± 0.96 vs. 0.52 ± 0.83, *p* < 0.05) (Fig. 7). COMP-Ang1 could be concluded to improve nerve function after nerve repair.

### Interaction Between Angiogenesis and Nerve Regeneration (*In Vitro*)

#### Morphological and Quantitative Evaluation of DRG Co-cultures

**Figure 8:**
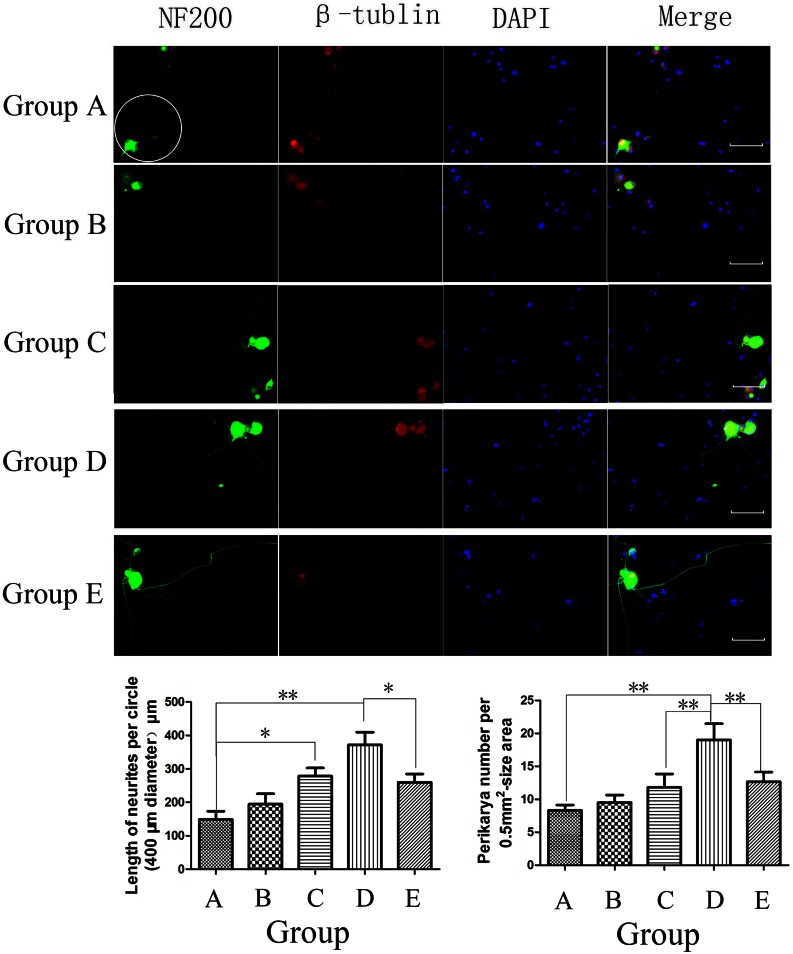
Morphologies of DRGs under various culture conditions (group A: DRGs alone, group B: DRGs with 100 ng/mL COMP-Ang1, group C: ECV304/DRGs co-culture, group D: ECV304/DRGs co-culture with 100 ng/mL COMP-Ang1, group E: ECV304/DRGs co-culture with 100 ng/mL COMP-Ang1 + 250 nM Tie-2 kinase inhibitor S157701). As an index of neurite outgrowth, the length of NF-positive neurites of first and higher orders was measured within a 400-*μ*m diameter circle around the perikaryon (see circle in group A). Significantly longer neurite length in ECV304/DRGs co-culture and ECV304/DRGs co-culture with COMP-Ang1. Number of NF-positive perikarya determined in 0.5-mm^2^ area per culture well. Significantly higher perikarya number in the ECV304/DRGs co-culture with COMP-Ang1 than in the ECV304/DRGs co-culture. The Tie-2 kinase inhibitor S157701 dramatically decreased the neurite length and number of perikarya in the ECV304/DRGs co-culture with COMP-Ang1. *p < 0.05 and **p < 0.01. Error bars correspond to the mean ± SD. Scale bars = 200 *μ*m.

**Figure 9:**
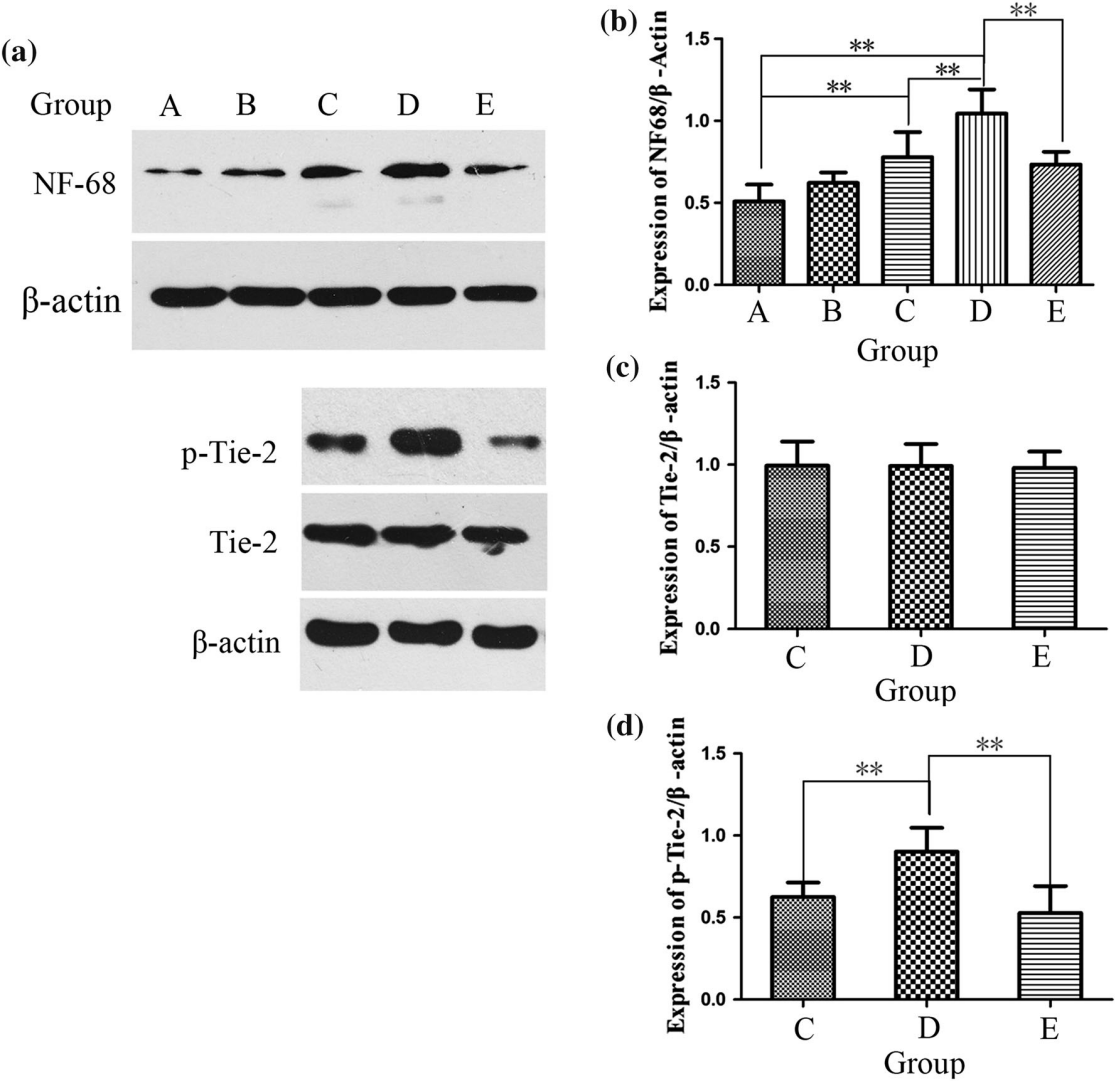
Western blot analysis of NF68, Tie-2 and phosphorylated-Tie-2 in various conditions (group A: DRGs alone, group B: DRGs with 100 ng/mL COMP-Ang1, group C: ECV304/DRGs co-culture, group D: ECV304/DRGs co-culture with 100 ng/mL COMP-Ang1, group E: ECV304/DRGs co-culture with 100 ng/mL COMP-Ang1 + 250 nM Tie-2 kinase inhibitor S157701). NF-immunoreactive bands (gray value analysis) confirmed that COMP-Ang1 administration significantly increased NF68 in ECV304/DRGs co-culture after the application of COMP-Ang1 (a, b). Tie-2 did not differ among these groups, but the level of p-Tie-2 was higher than that in the ECV304/DRGs co-culture (a, d). However, p-Tie-2 dramatically decreased when the phosphorylation of Tie-2 was blocked (a, d), which further decreased NF68 expression (a, b). *p < 0.05 and **p < 0.01. Error bars correspond to the mean ± S.D. Scale bars = 200 *μ*m.

The bar chart for group A shows that DRGs had sparse NF200-positive neurite outgrowth, fewer perikarya, and slightly reduced NF68 protein levels; groups A and B did not significantly differ (Figs. 8 and 9). When DRGs were co-cultured with ECV 304, the neurons showed promising neurite elongation and increased NF68 expression compared with group A, 287.00 ± 61.58 vs. 164.8 ± 50.13 *μ*m (*p* < 0.05) and 0.77 ± 0.16 vs. 0.54 ± 0.23 (*p* < 0.05), respectively (Figs. 9a and 9b). Though more perikarya could be observed in group C (Fig. 8), groups A and C did not significantly differ. The addition of COMP-Ang1 to the co-culture system increased the perikarya and NF68 protein level in group D compared with group C, 19.00 ± 6.06 vs. 11.83 ± 4.92 (*p* < 0.05) and 1.06 ± 0.15 vs. 0.77 ± 0.15 (*p* < 0.05), respectively. However, neurite length did not significantly differ between groups C and D (Fig. 8). In group E, in which a Tie-2 kinase inhibitor was used to inhibit Tie-2 function, neurite length, perikarya counts and NF68 expression dramatically decreased compared with group D, 259.67 ± 25.04 vs. 372.00 ± 92.16 *μ*m (*p* < 0.05), 12.67 ± 3.61 vs. 19.00 ± 6.06 (*p* < 0.05), and 1.06 ± 0.15 vs. 0.73 ± 0.08 (*p* < 0.05), respectively (Fig. 8). These results imply that p-Tie-2 likely plays a key role in the COMP-Ang1-mediated acceleration of neurite growth in the ECV304/DRGs co-culture system.

#### Mechanism Analysis of COMP-Ang1

The western blot data of the Tie-2/p-Tie-2 level indicate that the expression of Tie-2 did not significantly differ between groups C and D (0.99 ± 0.14 vs. 0.97 ± 0.13, *p* > 0.05) (Figs. 9a and 9c), but the level of p-Tie-2 was higher in group D than in group C (0.63 ± 0.09 vs. 0.90 ± 0.14, *p* < 0.05) (Figs. 9a and 9d). When the phosphorylation of Tie-2 was blocked with a Tie-2 kinase inhibitor in group E, the level of p-Tie-2 dramatically decreased compared with group D (0.90 ± 0.14 vs. 0.53 ± 0.16, *p* < 0.05), which subsequently decreased the expression of NF68 (1.04 ± 0.15 vs. 0.73 ± 0.08, *p* < 0.05).

## Discussion

The close association of peripheral nerves and blood vessels is an anatomical and functional relationship that has been known for centuries but has not been well described in a molecular and developmental context. Termed “neurovascular congruence,” this association stipulates that a growing nerve follows specific guidance cues produced by the vasculature to ultimately reach its appropriate targets.^20^ Consequently, blood supply is very important for peripheral nerve grafts.

Previous studies demonstrated that moderate neovascularization directly accelerates SC proliferation,^35^ improves vessel wall permeability,^3^ and subsequently promotes nerve regeneration,^24^,^33^ provided that the invasion of obstructive granular tissue is controlled to a low level.^2^,^4^ Conversely, a variety of neurotrophic factors were continuously transported to provide nutrition to SCs, while the membrane established an ideal substrate for nerve regeneration.^15^ The most exhaustively studied angiopoietins are Ang1 and Ang2. Ang1 is a critical player in vessel maturation, and it mediates the migration, adhesion and survival of ECs. Ang2 disrupts the connections between the endothelium and perivascular cells and promotes cell death and vascular regression. However, Ang2 also promotes neovascularization in conjunction with VEGF. Thus, VEGF, Ang1, and Ang2 may play complementary and coordinated roles in airway angiogenesis and microvascular remodeling, and therapeutic intervention may reverse these structural changes.^27^ Many studies focused on the therapeutic implications of targeting these angiogenic factors. Concerning the effect of VEGF on vascular formation, VEGF administration reportedly results in leaky, immature, and unstable vessels.^34^ In comparison, Ang1 is an angiogenic factor that plays important roles in the stabilization and maturation of blood vessels during angiogenesis.^42^ Ang1 also counteracts VEGF-induced inflammation in ECs while having an additive effect on vessel formation.^10^ Ang1 is a critical angiogenic factor for vascular maturation and enhances VEGF-induced angiogenesis in a complementary manner.^16^

Null mutation studies showed that Ang1 and its receptor Tie-2, a transmembrane tyrosine kinase uniquely expressed by ECs, are essential to developmental angiogenesis and are anti-apoptotic and neurotrophic to the neurons of the central and peripheral nervous system *in vitro*.^30^,^31^ The bifunctional trophic action of Ang1 on both blood vessels and nerve fibers suggest that Ang1 may have a wider therapeutic range, including beneficial effects in the treatment of nerve injury. COMP-Ang1 more potently phosphorylates the Tie-2 receptor than native Ang1.^7^,^28^,^32^ Thus, it may be more effectively involved in EC migration, pericyte recruitment and the formation, remodeling and maturation of blood vessels.^41^ Moreover, it can facilitate neurogenesis, thereby coordinating the healing injured nerve fibers and endoneural microvessels.^5^,^35^,^45^ Studies show that Akt and p38 MAPK phosphorylation are important downstream mediators of COMP-Ang1 and are involved in angiogenesis and axon growth.^14^,^32^ COMP-Ang1 induces the phosphorylation of Akt (Ser 473) and p38 MAPK (Thr180/Tyr182),^32^ suggesting that the angio- and neurotrophic actions of COMP-Ang1 in sciatic nerves involve the phosphorylation of Akt and p38 MAPK upon the Tie-2 receptor.

The *in vivo* experiments showed that VF (day 7 and day 14), VI (day 7) and NF200 IOD (day 14, 21, and 28) were higher in the ACNG + COMP-Ang1 group than in the ACNG group. After 14 days, some NF200-positive fibers were sporadically distributed in the ACNG + COMP-Ang1 group, while their expression was low in the ACNG group. Even though the number of NF200-positive fibers increased in both groups thereafter, the fluorescence remained stronger in the ACNG + COMP-Ang1 group than in the ACNG group. Taken together, the FG retrograde labeling, gastrocnemius atrophy, ultrastructure analysis, sciatic function and toluidine blue staining analysis (Figs. 4, 5, 6, and 7) clearly demonstrate the relationship between neovascularization and nerve regeneration. The results reveal that COMP-Ang1 increased the level of FG in the spinal cord, which suggested that the reverse transport of DRGs and motor neurons was improved. Moreover, decreased gastrocnemius atrophy, better sciatic function recovery and a well-organized neural architecture were also evident in the ACNG + COMP-Ang1 group after surgery, which indicate that COMP-Ang1 effectively promotes angiogenesis and axon regeneration. Here, we hypothesize that the following two pathways facilitate the beneficial effects of COMP-Ang1 on ACNG:

1. The promotion of neovascularization into the ACNG, which directly accelerates SC proliferation. The *in vivo* experiment verified that COMP-Ang1 may accelerate vessel formation to improve the nerve regeneration of the ACNG. At 14 days, the 3D vascular study clearly showed that vessels had already recanalized in the ACNG + COMP-Ang1 group but not in the ACNG group. Moreover, the blood supply was larger and more abundant in the ACNG + COMP-Ang1 group throughout the experiment. An anatomical study showed that the density of capillaries in the ganglion capsules of dorsal root ganglions and peripheral nerves was high. These capillaries are highly permeable to various compounds, which may be indispensable to maintain the normal function of nerve tissue.^1^ Furthermore, they may provide directional information for nerves to reach their target organs.^12^,^18^ Therefore, the early neovascularization of acellular nerve allografts may overcome a major current challenge in the nerve bioengineering field. Our investigation showed that COMP-Ang1 can accelerate the early neovascularization of nerve grafts, which not only aids the early invasion of inflammatory cells and removes fragments at the site but also imports numerous factors to nourish the injured nerve.^35^
2. Direct anti-apoptotic and neurotrophic effects on neurons. Although we did not carry out this part in this paper, Kosacka *et al*.^30^,^31^ found that Ang1 directly affects the axonal outgrowth of sensory neurons *in vitro*. When the Tie-2 receptor was blocked with the anti-Tie-2 antibody, neurite outgrowth was severely impeded, which supported that COMP-Ang1 may have anti-apoptotic and neurotrophic effects on neurons.^30^ We show that NF68 expression was increased in the ECV304/DRGs co-culture compared with DRG monoculture, and COMP-Ang1 significantly increased this effect in the co-culture system. Despite the lack of obvious differences in Tie-2 between the two co-culture systems described above, COMP-Ang1 significantly increased the expression of p-Tie-2 in the ECV304/DRGs co-culture system. Tie-2 kinase inhibitor did not change the expression of Tie-2 in the ECV/DRGs co-culture system treated with COMP-Ang1. However, the phosphorylation of Tie-2 was blocked, and p-Tie-2 expression dramatically decreased, which consequently decreased the expression of NF68. These results indicate that the difference in NF68 levels correlated with the difference in p-Tie-2 levels, which signified that the activation of Tie-2 may play an underlying role in the neurite outgrowth of DRGs.

**Figure 10:**
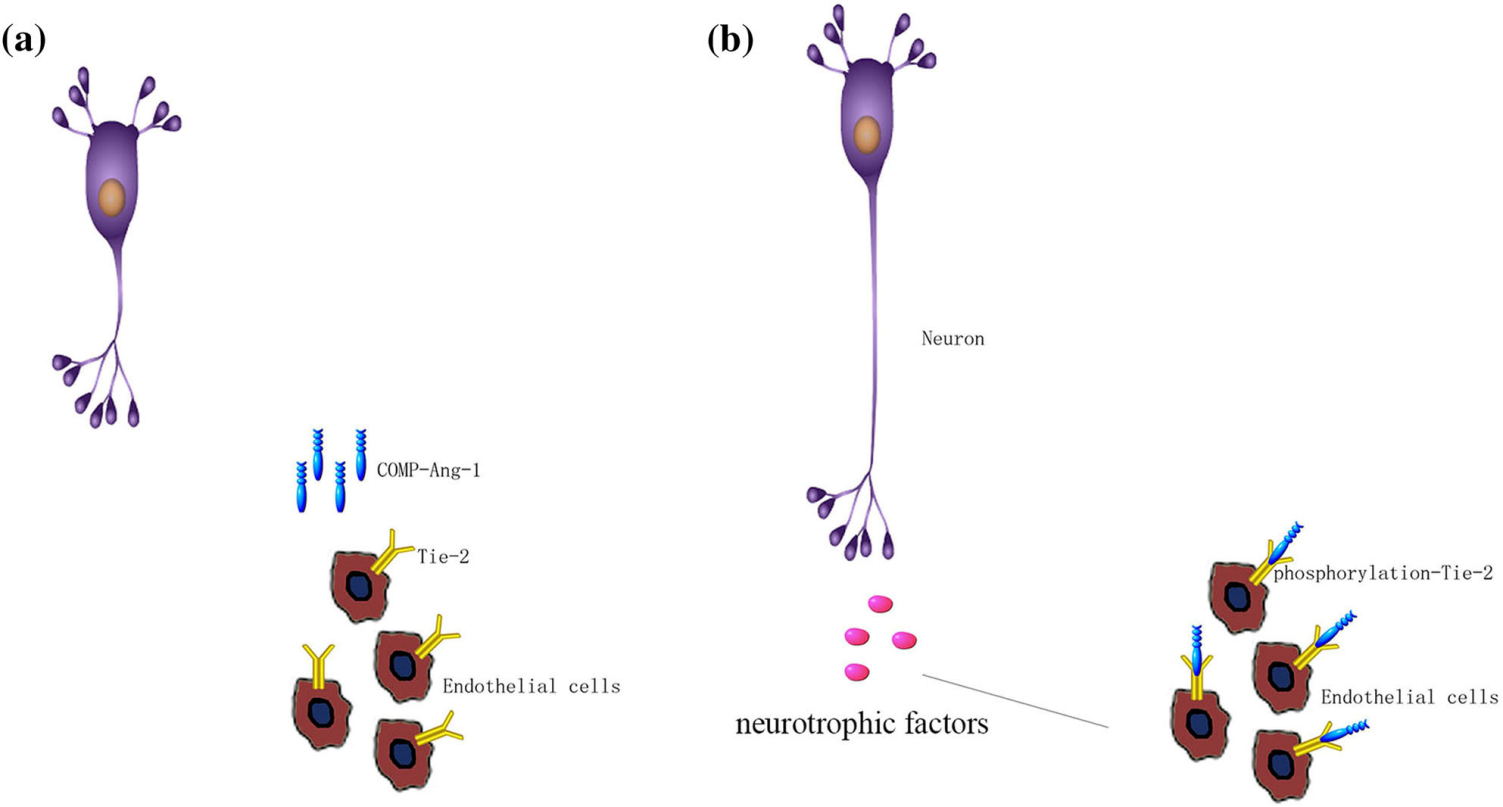
COMP-Ang1 modulates the phosphorylation of Tie-2 in ECs, and ECs secrete trophic factors to affect the regeneration of neurons (a and b).

Although previous studies have demonstrated the direct effects of Ang1,^29^,^30^ its indirect influence was not as obvious. We found that DRGs have longer neurites and are more viable when co-cultured with ECs (ECV304) than when cultured alone and that COMP-Ang1 could significantly enhance this effect. As shown in previous studies, ECs and neural cells exhibit several layers of cross-talk that affect neurogenesis and neural cell fate.^5^ Gibran *et al*. have demonstrated that ECs can produce NGF, which can prompt neurite outgrowth.^19^ In addition, ECs can release neurogenic factors, such as brain-derived nerve factor (BDNF), to promote nerve repair.^35^ Here, we hypothesize that ECs may release various growth factors that significantly impact the nerve regeneration of the COMP-Ang1-targeted Tie-2 pathway (Figs. 10a and 10b). Thus, other neurotrophic factors secreted by ECs, such as NGF and BDNF, may play an important role in neurogenesis.^37^

In summary, this study included *in vivo* and *in vitro* experiments to investigate the role of COMP-Ang1 in improving ACNG efficacy when repairing peripheral nerve defects. It supported that COMP-Ang1 improved the neovascularization of ACNG to promote nerve regeneration *in vivo*. A series of experiments to elucidate the mechanism of COMP-Ang1 showed that COMP-Ang1 was angiogenic and could enhance neurogenesis when neural cells (DRGs) were co-cultured with ECs *in vitro.* However, several limitations of this study should be noted: (1) we failed to establish a 3D ECV304/DRGs co-culture system to detect the role of COMP-Ang1/Tie-2 in angiogenesis; (2) we confirmed that COMP-Ang1 enhanced neovascularization but could not detect specific neurotrophic factors due to neovascularization *in vivo* and *in vitro*; (3) we initially selected NF200 for the fluorescence staining and western blot analyses. However, the NF200 bands in the western blot analysis were not as clear as desired for practical applications. Therefore, we selected NF68 antibodies for the western blot analysis based on a literature search.^30^,^31^,^36^ (4) The defect length was insufficient because the animals were small. Our anatomical studies indicated that the maximal length of the sciatic nerve that needed to be cut in rats was approximately 17 mm. Because nerve tissue can retract immediately after being cut due to its elasticity and all ACNGs need to be trimmed after extraction, a 17-mm nerve graft shortens to only 12 mm. Though a long defect in a human is defined as having a minimum length of 30 mm, nerves in rats were often shorter than 17 mm in the lower extremities. Larger animals, such as rhesus monkeys, which are more closely related to humans than rats, could be used as feasible models to verify the efficacy of COMP-Ang1 in repairing long and large nerve defects. We will further investigate the interaction between neovascularization/angiogenesis and axon regeneration and answer the above questions in future studies.
